# Stability of *Lactobacillus rhamnosus* GG incorporated in edible films: Impact of anionic biopolymers and whey protein concentrate

**DOI:** 10.1016/j.foodhyd.2017.04.014

**Published:** 2017-09

**Authors:** Christos Soukoulis, Solmaz Behboudi-Jobbehdar, William Macnaughtan, Christopher Parmenter, Ian D. Fisk

**Affiliations:** a Environmental Research and Innovation, Luxembourg Institute of Science and Technology (LIST), 5. Avenue des Hauts-Fourneaux, L-4362, Esch sur Alzette, Luxembourg; b Division of Food Sciences, School of Biosciences, University of Nottingham, Sutton Bonington Campus, Loughborough, LE12 5RD, Leicestershire, United Kingdom; c Nottingham Nanotechnology and Nanoscience Centre, University of Nottingham, University Park, Nottingham, NG7 2RD, United Kingdom

**Keywords:** Probiotic, Edible film, Alginate, Pectin, Carrageenan, Dairy protein

## Abstract

The incorporation of probiotics and bioactive compounds, via plasticised thin-layered hydrocolloids, within food products has recently shown potential to functionalise and improve the health credentials of processed food. In this study, choice of polymer and the inclusion of whey protein isolate was evaluated for their ability to stabalise live probiotic organisms. Edible films based on low (LSA) and high (HSA) viscosity sodium alginate, low esterified amidated pectin (PEC), kappa-carrageenan/locust bean gum (κ-CAR/LBG) and gelatine (GEL) in the presence or absence of whey protein concentrate (WPC) were shown to be feasible carriers for the delivery of *L. rhamnosus* GG. Losses of *L. rhamnosus* GG throughout the drying process ranged from 0.87 to 3.06 log CFU/g for the systems without WPC, losses were significantly reduced to 0 to 1.17 log CFU/g in the presence of WPC. Storage stability (over 25d) of *L. rhamnosus* GG at both tested temperatures (4 and 25 °C), in descending order, was κ-CAR/LBG > HSA > GEL > LSA = PEC. In addition, supplementation of film forming agents with WPC led to a 1.8- to 6.5-fold increase in shelf-life at 4 °C (calculated on the WHO/FAO minimum requirements of 6 logCFU/g), and 1.6–4.3-fold increase at 25 °C. Furthermore probiotic films based on HSA/WPC and κ-CAR/LBG/WPC blends had both acceptable mechanical and barrier properties.

## Highlights

• Whey protein concentrate (WPC) inclusion enhanced survival during drying and storage.
• WPC inclusion reduced water vapour permeability and T_g._
• Cell viability was greatest in pectin/WPC films during drying.
• Cell viability was greatest in composite carrageenan/locust bean gum/WPC films during storage.

## Graphical abstract

*L. rhamnosus* GG survival in edible films – the interrelation between the physical and optical film properties and *L. rhamnosus* GG survival during storage.

**Figure:**
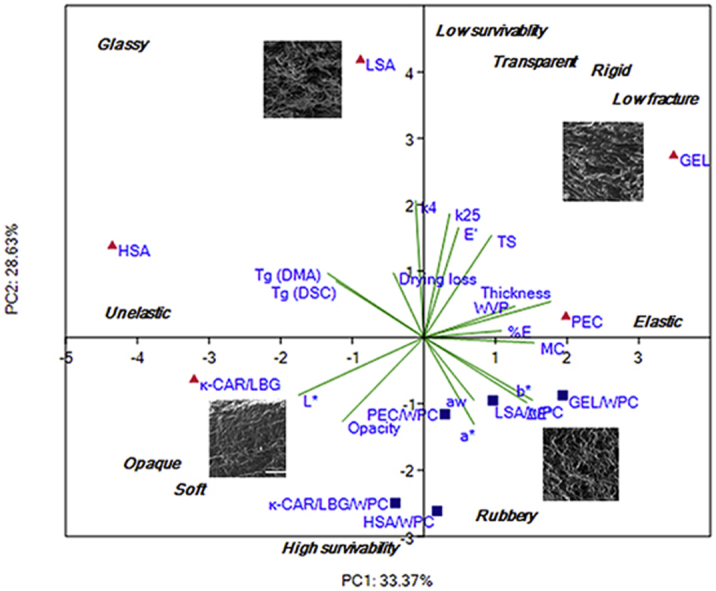

## 1 Introduction

According to the FAO/WHO probiotics are “viable microorganisms which when administered in adequate amounts (>10^6^–10^7^ CFU/g of ingested product) may confer health benefits to the human host”. Reported health-associated benefits of probiotics include modulation of the gastrointestinal system, reduction in rotavirus and antibiotic induced diarrhoea, stimulation of the immune system and reduction of lactose intolerance and irritable bowel symptoms (Saad, Delattre, Urdaci, Schmitter, & Bressollier, 2013). Due to the sensitivity of probiotics to common processing conditions such as heat treatment, low pH environments, high osmotic pressure and high redox potentials, the design of effective physicochemical barriers to stabilise the organisms is essential to their full commercial exploitation in a wide range of food categories (Burgain et al., 2011, Jankovic et al., 2010, Meng et al., 2008). Anhydrobiotics technology i.e. the encapsulation of living cells in low moisture (glassy) matrices fabricated via spray or freeze drying, remains to date the most popular approach to ensure maximal viability of probiotics (Behboudi-Jobbehdar et al., 2013, Burgain et al., 2011, Meng et al., 2008, Soukoulis et al., 2014a, Tripathi and Giri, 2014). Nevertheless, the use of edible films (plasticised thin layered biopolymer structures) to embed viable probiotic cells is increasingly being studied (Gialamas et al., 2010, Kanmani and Lim, 2013, López de Lacey et al., 2014, López de Lacey et al., 2012, Romano et al., 2014, Soukoulis et al., 2014c, Soukoulis et al., 2016). Edible films have the potential to stabilise food structures at multiple scale lengths whilst creating bespoke structures (enhanced mechanical properties, prolonged shelf-life, maintenance of structural integrity) and be used to deliver nutritional enhancements through probiotic inclusion. On the downside, inclusion of plasticisers may increase the lethality of entrapped bacterial cells due to osmolysis, inability to completely repress the cellular metabolic activity and increased exposure to oxygen, but are essential for the formation of edible films. To overcome this, the inclusion of compounds that scavenge free radicals, promote cells adhesion properties and suppress the matrix's glass transition temperature are often proposed (Burgain, Gaiani, Francius, et al., 2013). Edible films could offer significant benefits for intermediate moisture foods (IMF) when compared to conventional dehydrated microcarriers, this is mainly due to their ability to retain their physical state and biological activity throughout IMF storage, where dehydrated microcarriers, as opposed to edible films, in most cases experience structural collapse due to physical state transitions (glassy to rubbery) resulting in reduced cell viability. Hence, a vast number of applications have been investigated for edible film and coating technologies, these include bakery products, fishery products, dried fruits, olives, cereal bars (Altamirano-Fortoul et al., 2012, De Prisco and Mauriello, 2016, López de Lacey et al., 2012, López de Lacey et al., 2014, Soukoulis et al., 2014a, Tavera-Quiroz et al., 2015).

To understand the potential of edible films as vehicles for probiotics inclusion, parameters such as the biopolymer and plasticiser type and amount, the presence of oxygen scavenging agents and prebiotics have been recently evaluated (Gialamas et al., 2010, Kanmani and Lim, 2013, López de Lacey et al., 2014, Piermaria et al., 2015, Romano et al., 2014, Soukoulis et al., 2014, Soukoulis et al., 2014b, Soukoulis et al., 2016). In a previous work, we demonstrated that the inclusion of *L. rhamnosus* GG in edible films, comprising whey protein concentrate and sodium alginate, assisted bacterial cells to withstand heat and osmotic stress upon bread production and storage whereas it also enhanced their survival throughout ingestion and gastrointestinal passage (Soukoulis et al., 2014). In the present work, we aim to further investigate the technological feasibility of edible films comprising selected biopolymers with established good film forming properties (namely low esterified amidated pectin (PEC), low (LSA) and high (HSA) viscosity sodium alginate, porcine skin gelatine (GEL) and kappa-carrageenan/locust bean gum (κ-CAR/LBG)), in the presence or absence of whey protein concentrate (WPC) as potential vehicles for *L. rhamnosus* GG. Selection of the biopolymers and compositional design of the edible film forming solutions was based on previous formulations for effective films and are constrained by practical and biopolymer specific requirements. Both protein and polysaccharide based films and binary films containing two polysaccharides are included to expand the range of the study (Galus and Lenart, 2013, Martins et al., 2012, Ramos et al., 2012, Rivero et al., 2010). Ultimately the aims was to explain the interplay between the survivability of *L. rhamnosus* GG and the structural and physicochemical properties of the embedding biopolymer substrate.

## 2 Materials and methods

### 2.1 Materials

For the purposes of this work a *Lactobacillus rhamnosus* GG strain (E-96666, VTT, Espoo, Finland) of established probiotic functionality was used. Low ester content (<50%) amidated pectin (LM-101 AS, Genu^®^, CPKelco, UK), low viscosity sodium alginate (LFR5/60, Protanal^®^, 65–75% guluronic acid units, 25–35% mannuronic acid, units, 35–60 kDa, Drammen, Norway), high viscosity sodium alginate (RF6650, Protanal^®^, 45–55% guluronic acid units, 45–55% mannuronic acid, units, ∼100 kDa, Drammen, Norway), locust bean gum (Sigma Aldrich, UK), kappa-carrageenan (Sigma Aldrich, UK) and bovine skin gelatin B (Sigma Aldrich, UK) were used as film forming agents. Whey protein concentrate (81 ± 2% whey protein, 9% lactose, Lacprodan^®^ DI-8090) was used as a co-structuring component, glycerol (97% purity, Sigma Aldrich, UK) was used as the plasticiser.

### 2.2 Preparation of the film forming solutions

Ten film forming solutions were prepared by dispersing the biopolymers and WPC (as listed in Table 1) in distilled water at 25 °C under agitation for 1 h. Then, glycerol accounting for the 50% (w/w) of the film forming agent total solids was added and the obtained biopolymer aliquots were heated to 80 °C for 30min. Heat treatment assisted the full desolution and hydration of the biopolymers, induced whey protein denaturation (>95%) and reduced residual microbial load. Eventually, the film forming solutions were cooled to 37 °C to be inoculated with *L. rhamnosus* GG.

### 2.3 Stock culture preparation and growth conditions of L. rhamnosus GG

Stock culture preparation of *L. rhamnosus GG* was carried out according to the procedure as previously described by Soukoulis et al. (2014a). Six frozen culture beads were placed in MRS broth (Oxoid Ltd., Basingstoke, UK) and incubated at 37 °C (48 h) under anaerobic conditions in plastic jars containing AnaeroGen^®^ (Oxoid Ltd., Basingstoke, UK). The final broth was transferred under aseptic conditions into 50 mL sterile centrifuge tubes (Sarstedt Ltd., Leicester, UK) and centrifuged at 3000 g for 5 min. Pellets were washed twice with phosphate buffer saline (PBS), Oxoid Ltd. Basingstoke, UK.

### 2.4 Preparation and storage of the probiotic edible films

Film forming solutions (100 mL) were inoculated with three pellets (corresponding to ca. 10 logCFU/g of film forming solution, expressed in a dry basis) and successively degassed using a vacuum pump at 40 °C for 10 min. Then, 30 mL of the aliquots were aseptically transferred using a serological pipette to sterile petri dishes (inner diameter 15.6 cm; polystyrene; 101VR20, Sarstedt Ltd., Leicester, UK). The cast solutions were dried for 24 h in a ventilated incubator at 37 °C and ca. 50% RH (Sanyo Ltd., Japan). After air drying, the probiotic edible films were peeled off intact from the petri dishes and conditioned either at room temperature (25 °C) or chilling conditions (4 °C) for microbiological testing under controlled relative humidity conditions (ca. 54 and 59% RH respectively) using a saturated magnesium nitrate solution (Sigma Aldrich, Basingstoke). Separate systems conditioned for at least three days at 25 °C and 54% RH were used for physicochemical, mechanical and structural characterisation.

### 2.5 Enumeration of the bacteria

One mL of the probiotic film forming solutions was suspended in 9 mL sterile PBS and vortexed for 60s to ensure adequate mixing. For the recovery of *L. rhamnosus* GG from the probiotic edible films the method described by (Soukoulis et al., 2014a, Soukoulis et al., 2014b, Soukoulis et al., 2014c) was adopted. Specifically, 1 g of the film containing *L. rhamnosus GG* was mixed with 9 mL of PBS and vortexed for 2 min to ensure sufficient dissolution of the film. Enumeration of the bacteria was performed in triplicate following the standard plating methodology (Champagne, Ross, Saarela, Hansen, & Charalampopoulos, 2011) and the total counts of the viable (TVC) bacteria were expressed as log colony forming units per gram (log CFU/g) by taking into account the density (g/mL) of the film forming solutions calculated gravimetrically.

The survival rate of the bacteria throughout the air drying of the film forming solutions was calculated according to the following equation:

(1) $$\%\text{ viability}=100\times \frac{\text{N}}{{\text{N}}_{0}}$$

where N_0_ and N represent the number of viable bacteria (expressed by total solids amount at the beginning and end of the air drying process respectively).

*L. rhamnosus* GG inactivation upon storage was expressed as the logarithmic value of the relative viability fraction (log N/N_0_). Viability was fitted to a first order reaction kinetics model as described by the formula:

(2) $$\text{log}\;{\text{N}}_{\text{t}}=\text{log}\;{\text{N}}_{\text{0}}-{\text{k}}_{\text{T}}\text{t}$$

where N_0_, represents the initial number of the viable bacteria and N_t_ the number of viable bacteria after a specific time of storage (CFU/g), t is the storage time (day), and k_T_ is the inactivation rate constant (logCFU·day^−1^) at temperature, T (°C).

### 2.6 Moisture content and water activity

Residual water content was calculated according to AACC method 44-1502. Water activity of the edible films after preconditioning at 54% RH for 72 days was determined using an AquaLab water activity meter (AquaLab, 3 TE, Decagon, USA).

### 2.7 Scanning electron microscopy (SEM)

A small film specimen was carefully deposited onto carbon tabs (Agar Scientific, Stansted, UK) and coated with carbon (Agar turbo carbon coater) to improve conductivity. Scanning electron microscope analysis (SEM) was performed on a FEI Quanta 3D 200 dual beam Focused Ion Beam Scanning Electron Microscope (FIB-SEM). The images were acquired using secondary electron imaging at an accelerating voltage of 5–15 kV.

### 2.8 Thickness measurement

A digital micrometer (Mitutoyo, Tokyo, Japan) was used for the measurement of the thickness (mm) of the probiotic edible films. Eight measurements were taken from different parts of the films.

### 2.9 Water vapour permeability

Water vapour permeability (WVP) of the probiotic edible films was determined gravimetrically. Samples were placed between two rubber rings on the top of glass cells containing silica gel (0% RH) to 1/6 of cell height, exposed film area was 2.9 × 10^−3^ m^2^. The glass cells were transferred to a ventilated chamber maintained at 100% RH (pure water) and 25 °C, water vapour pressure difference is 3169 Pa. WVP was calculated according to the formula:

(3) $$\text{WVP}=\;\frac{\text{Δ}\text{m}\cdot \text{e}}{\text{A}\cdot \text{Δ}\text{t}\cdot \text{Δ}\text{p}}$$

where: WVP = water vapour permeability (g.mm.m^−2^. d^−1^. kPa^−1^) Δm/Δt = the moisture uptake rate (g/d) from silica gel, A = the film area exposed to moisture transfer (m^2^), e = the film thickness (m), and Δp = the water vapour pressure difference between the two sides of the film (Pa).

### 2.10 Colour characteristics and opacity

Colour characteristics of the edible films were determined using a Hunterlab (Reston, USA) colorimeter. The CIELab colour scale was used to measure the L* (black to white), a* (red to green), and b* (yellow to blue) parameters. Film samples (2 cm × 2 cm) were carefully deposited on a standard white tile (L* = 92.59, a* = -0.78, b* = 0.67).

Opacity measurements were made according to the method described by Núñez-Flores et al. (2012). Film samples were cut into rectangles (0.7 × 1.5 cm) and placed carefully on the surface of the plastic cuvette and on the spectrophotometer cell after calibration with an air blank sample. Absorbance at 550 nm (A_550_) was measured using a UV-VIS spectrophotometer (Jenway Ltd., UK) and film opacity was calculated according to the formula:

(4) $$\text{Opacity}=\;\frac{{\text{A}}_{550}}{\text{thickness}}$$

where: thickness is expressed in mm.

### 2.11 Mechanical characterisation

Mechanical characterisation (tensile strength (TS), elongation percentage (% E) at break, and Youngs modulus (E), calculated as the slope of the linear region of the stress-strain curve) of the films was conducted using a TA-XT2i texture analyser (Stable Micro Systems Ltd, Surrey, UK). Pre-conditioned edible films (54% RH, 25 °C for 72 h), cut in 20 × 80 mm rectangular shapes were placed between the tensile grips giving a grip separation distance of 50 mm. For tensile tests a 5 kg load cell was used with a cross-head speed of 1 mm/s. The following properties were calculated from the stress – deformation curves:

(5) $$\text{TS}=\frac{{\text{F}}_{\text{max}}}{\text{A}}$$

(6) $$\%\;\text{E}=100\times \;\frac{\text{L}}{{\text{L}}_{0}}$$

(7) $$\text{E}=\;\frac{\Delta \varsigma }{\text{Δ}\varepsilon }$$

where: F_max_ = the force at break (N), A = the film cross-sectional area (mm^2^), L_0_ = the initial film length (mm), L_t_ = the film length at time t (linear region) (mm), L = the film length at break (mm), strain = *ε* = (L_t_-L_o_)/L, stress = σ = F/A (MPa).

### 2.12 Dynamic mechanical analysis (DMA)

The dynamic mechanical measurements were carried out using a Perkin Elmer DMA8000 (Coventry, UK) operating in the tension mode. The film samples were prepared and then cut in 0.5 × 2 cm rectangular strips and conditioned at 54 ± 1% RH and 25 ± 1 °C for 72 h before analysis. The film samples were clamped in the tension geometry attachment and analysis was conducted by heating the samples at 2 °C min^−1^ from −80 to 180 °C. From experimental data, the storage modulus (E′), loss modulus (E″) and tanδ (E″/E′) were calculated, glass transition temperature (T_g_) was defined as the peak value of tanδ. All analyses were conducted in duplicate.

### 2.13 DSC measurements

A Mettler Toledo DSC823 (Leicester, UK) was used for the measurement of the glass transition temperature of the edible films. A small amount of plasticised pre-weighed edible film (6–10 mg) was placed in a high-pressure, stainless steel pan and subjected to the following cooling – heating protocol: 1) cool from 25 to −120 °C at 50 °C min^−1^, 2) hold isothermally at −120 °C for 10 min, 3) heat from −120 to 200 °C at 5 °C min^−1^ and 4) cool from 200 to −120 °C at 50 °C min^−1^ 5) hold isothermally at −120 °C for 10 min, 6) heat from −120 to 200 °C at 5 °C min^−1^ and 7) cool from 200 to 25 °C at 50 °C min^−1^. The onset (T_g,on_) and midpoint glass transition temperature (T_g,mid_) were calculated from the second heating step.

### 2.14 Statistical analyses

Two-way ANOVA joint with Duncan's post hoc means comparison (p < 0.05) test was performed to evaluate the main effects of the investigated factors (film forming agent, addition of WPC) on the microbiological, physicochemical and mechanical data. Repeated measures ANOVA was used to evaluate the impact of storage time on survival rates of *L. rhamnosus* GG. Principal component analysis (PCA) and Pearson's correlation tests were carried out to investigate the interrelationships of the film's compositional profile and their respective microbiological, physicochemical and mechanical properties. All statistical treatments were performed using the MINITAB release 16 statistical software (Minitab Inc., PA, USA).

## 3 Results and discussion

### 3.1 Survival of *L. rhamnosus* GG throughout drying process

**Fig. 1:**
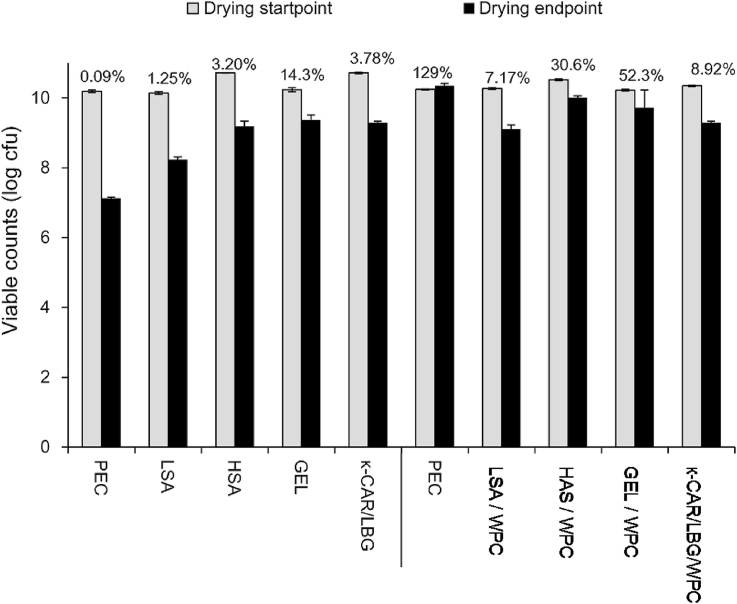
Changes in the total viable counts of *L. rhamnosus* GG during the film forming dehydration process. (error bars indicate ± 1 SD, percentages indicate percentage retention/increase after drying).

**Fig. 2:**
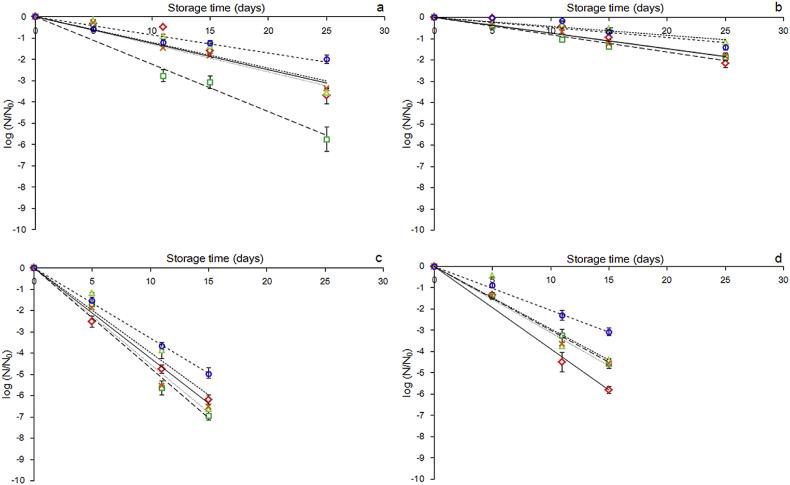
Inactivation curves of *L. rhamnosus* GG embedded in edible films preconditioned at 54% RH and stored either at chilling (4 °C, a,b) or ambient temperature conditions (25 °C, c,d) up to 25 and 15 days respectively, without (a,c) and with WPC (b,d). (PEC dark solid line; LSA solid dashed line; HSA dotted line; GEL light solid line; K-CAR/LBG light dashed line).

Edible films are a promising route for the control and enhancement of functional and technological aspects of processed food (Falguera et al., 2011, Ramos et al., 2012). Edible film based strategies could also be used for the delivery of bioactive compounds and beneficial cells into staple food items. The chemistry of the film and film forming procedure is of paramount importance as it is directly associated with bacterial survival post-processing (exposure to low pH and low redox environments, presence of oxygen) and post-ingestion (exposure to digestive enzymes and bile salts, low pH). The TVCs of *L. rhamnosus* GG 1 h after inoculation of the film forming aliquots (10.2 ± 0.2 log CFU/g) showed no acute toxic effects of the biopolymer type or WPC on cell viability either during film production or over shelf life (Fig. 1, Fig. 2, Table 2) which is important to note as in our previous studies, we observed that cells belonging to the *L. rhamnosus* and *L. acidophilus* strains when injured due to osmotic and heat stress during film forming, exhibited a higher lethality throughout storage and under *in vitro* pre-absorptive digestion conditions (Soukoulis et al., 2014a, Yonekura et al., 2014).

Although there was no overall toxic effects on the survival of the *L. rhamnosus* GG throughout the air drying process (37 °C, 50% RH, 24 h) viability was significantly (p < 0.05) influenced by the compositional characteristics (hydrocolloid type, WPC addition) of the film forming solutions (Fig. 1), which is in agreement with the findings from our previous studies (Soukoulis et al., 2014, Soukoulis et al., 2014c, Soukoulis et al., 2016). As a general trend, polysaccharide based films (PEC, LSA, HSA and κ-CAR/LBG) exerted the highest cell lethality (96.2–99.9%, please note that numbers in Fig. 1 represent survival rates), compared to the one including protein (85.7%). On supplementation with WPC, a 2.4–10-fold increase in *L. rhamnosus GG* survivability was observed for film forming solutions comprising alginates, GEL and the κ-CAR/LBG binary blend, whilst interestingly in the case of PEC/WPC film forming systems *L. rhamnosus* GG underwent mild growth. Whilst monitoring water activity during the drying process (data not shown), it was observed that during the stage of constant rate drying (ca. 6 h) water activity was higher than the minimum threshold required for the growth of *Lactobacilli* (a_w,opt_ = 0.91) therefore favouring the growth of *L. rhamnosus* GG. During the falling rate drying stage, water evaporation gives rise to osmotic pressures that can induce osmolytic sub-lethal effects on bacterial cells. And if the temperature is sufficient, heat shock related cellular injuries may be also experienced by the bacterial cells, yet this is strictly dependent on the drying temperature. We believe that the stability, of the lack of stability is a function of the biopolymer chemistry, with certain biopolymers hampering osmolysis and inducing protection to heat shock sub-lethal effects via several mechanistic pathways including modulation of adhesion properties, scavenging free radicals, supplying micronutrients (e.g. free amino acids) and maintenance of the native physical state of cell membranes (Barriga and Piette, 1996, Burgain et al., 2013b, Deepika and Charalampopoulos, 2010, Fu and Chen, 2011, Ghandi et al., 2012, Tripathi and Giri, 2014). It may also be true that other intrinsic parameters such as the pH (pH_opt_ = 5.7, VTT, Espoo, Finland), low redox potential, and the surface tension of the substrate may modulate *L. rhamnosus* GG viability in the tested films by enhancement cell mobility and spreading. With regards the optimum pH for growth of L. rhamnosus, the low pH of the pectin film solution without WPC (pH 3.9–4.2) could explain the acute lethality observed in the pectin based systems, the pH of the alginate solution was higher at pH 5.4–5.7, the κ-CAR/LBG and GEL had comparable pH values of 6.3–6.7.

It has been previously reported that *L. rhamnosus* cells are negatively surface charged over a broad pH range (3-10) and therefore their adhesion properties are governed by either electrostatic interactions (with positively charged biopolymers or protonated side carbon chain groups) or more probably, for most of the anionic polysaccharides used in the present study, via hydrogen bonding (Deepika, Green, Frazier, & Charalampopoulos, 2009). In general, the polysaccharides we tested were negatively surface charged and possess no tensioactive properties and therefore bear no evident bacteria adhesion ability. Gelatine, is a predominantly negatively charged protein and is generally considered as having a modest tensioactive properties (surface tension ca. 50 dyn/cm) and has exposed hydrophobic groups that could promote bacteria adhesion via hydrophobic interactions. This may explain why gelatin (without WPC) is the most stable during air drying.

The addition of WPC was associated with a slight increase in the pH of the film forming solutions, this was most significant in the PEC/WPC system (pH 5.4–5.6). Furthermore, in recent comparative studies on milk protein adhesion properties, it was demonstrated that whey proteins possessed the highest adhesion properties with *L. rhamnosus* GG cells via electrostatic and hydrophobic binding (Burgain et al., 2013b, Burgain et al., 2013a). The peculiar behaviour observed in the PEC/WPC may also be attributed to phase separation between the pectin and whey protein forming localised microdomains enriched in either component (Tolstoguzov, 2003). It is therefore hypothesised that the buffering capacity of WPC in combination with water activity suitable for growth and its other intrinsic properties, phase separation and cellular adhesion may account for the enhanced survival rates of *L. rhamnosus* GG in the PEC-WPC system during drying.

Finally, biopolymer entanglement taking place via the physical entrapment of probiotic cells and retention of water in hydrogel interspaces may aid *L. rhamnosus* GG cells to maintain their native physical cell structure, this may explain the better performance of biopolymers with good hydrogel forming ability e.g. HSA and κ-CAR/LBG.

### 3.2 Microstructure of film cross-section

**Fig. 3:**
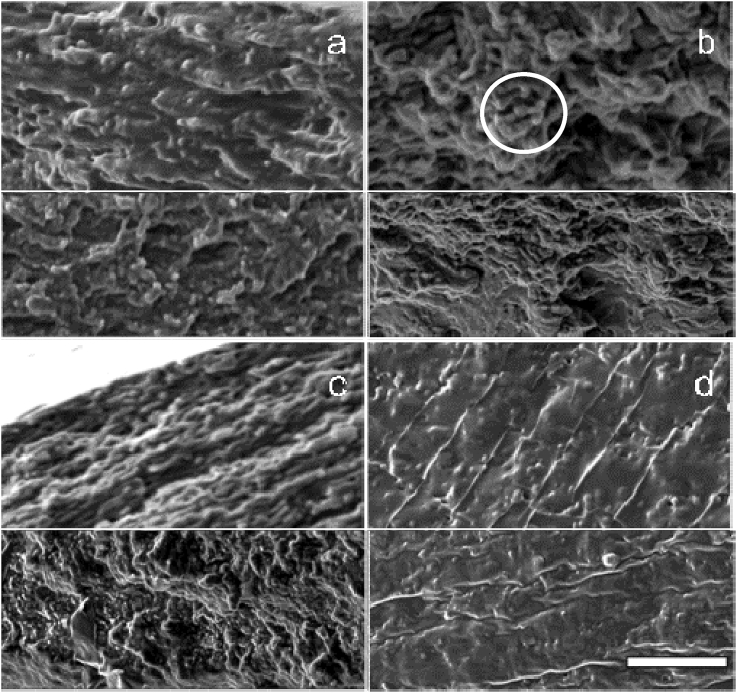
SEM micrographs of the probiotic hydrogel-based edible films cross section with (upper) and without (lower) WPC. (a): Pectin, (b): LV sodium alginate, (c): HV sodium alginate, (d): kappa-carrageenan/LBG-(8:2). Scale bar = 10 μm, the cells of L. rhamnosus GG embedded in the biopolymer matrices are tentatively marked with white circles.

Structural conformation, cross-sectional homogeneity and encapsulation efficiency of the probiotic cells was evaluated by focused ion beam scanning electron microscopy (Fig. 3). Corroborating our previous findings (Soukoulis et al., 2014b), FIB-SEM allowed the successful visualisation of the cells of *L. rhamnosus GG* embedded in the biopolymer matrices (see Fig. 3).

As illustrated in Fig. 3, the biopolymer type had a governing role on the development of the main microstructural aspects, with films fabricated with κ-CAR/LBG exhibiting the most compact structures, generally void of cracks, fissures or hollow micro-domains. On the contrary, the rest biopolymer samples had a reticular, honeycomb-like microstructure with bud-like protrusions; however, in all cases the films did not have a highly perforated structure suggesting the development of rather dense and tightly-packed biopolymer networks indicating good mechanical durability and barrier properties (Lacroix, 2009).

The addition of WPC (Fig. 2) did not modify the overall film structure; however, according to micrographs, the presence of whey proteins had an interplaying role with the film forming agent leading a more compact structure. In addition, whey proteins induced the formation of a finer and less coarse reticular structure similar to that observed in acid whey gels (van den Berg, Rosenberg, van Boekel, Rosenberg, & van de Velde, 2009). In the case of κ-CAR/LBG no detectable structural changes were identified on the addition of WPC.

### 3.3 Physical characteristics

As aforementioned, two distinct drying phases (data not shown) were verified throughout the film forming process: first, a constant drying rate (ranging from 285 to 310 min) and a falling drying rate (from 6 to 18 h). Equilibrium moisture contents for all films were achieved during the last 4 h of the drying process. No significant differences in the drying kinetics were observed and water evaporation rates during the constant rate drying phase ranged from 0.106 to 0.113 g min^−1^.

Residual moisture content of the films at the end of the drying process (before the RH preconditioning step), was significantly affected by the type of film forming agent and presence of whey proteins (Table 3). In general, the concentration, water holding capacity and structuring ability of the biopolymers, in conjunction with the type and amount of plasticising agents, have previously been proposed as being the major parameters affecting equilibrium moisture levels in edible films (Thakhiew, Devahastin, & Soponronnarit, 2010). PEC-based films exhibited the highest moisture content whilst HSA and κ-CAR/LBG the lowest, as high moisture contents samples also had high thicknesses and the greater solids contents is assumed to be due to this. The addition of WPC also resulted in a significant increase (p < 0.05) in equilibrium moisture content (ranging from ca. 5–110% for GEL and κ-CAR/LBG systems respectively) compared to the WPC-free films, although on an individual basis there was only a significant increase for the HSA and κ-CAR/LBG based films. Whey protein powders are well known for their very good water holding capacity compared to milk or caseinate powders; this is mainly to the ability of whey proteins to interact with water molecules via hydrogen bonding and to the hygroscopicity of lactose and salts present at residual levels in WPC (Kinsella, Fox, & Rockland, 1986).

HSA and κ-CAR/LBG based films (but not their WPC based analogues) were thinner than the PEC, LSA and GEL systems which presumably could be attributed to their lower total solids content. The average thickness of the films was not affected by WPC addition, although there was an increase in thickness in the HSA (0.04 → 0.09 mm) and κ-CAR/LBG (0.04 → 0.10 mm) based films which again could be due to the relative enhancement in total solids being greater.

### 3.4 Water vapour permeability (WVP)

**Fig. 4:**
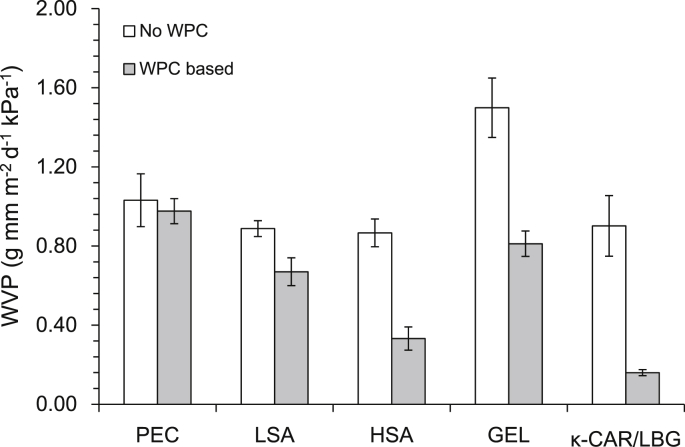
Water vapour permeability of probiotic edible films at ambient temperature (25 °C) and 100% RH gradient rate. (error bars indicate ± 1 SD).

Probiotic films containing LSA had lower WVP values compared to that of PEC and GEL, WVP of the probiotic edible films was significantly (p < 0.05) lower in the WPC based systems (Fig. 4) and WVPs of HSA and κ-CAR/LBG was strongly WPC dependent. In general, the affinity of a film forming agent to water may explain the differential permeability of the films, specifically the poor barrier properties of PEC and GEL films which could be attributed to their high water affinity which is also supported by the residual moisture data (Table 3). The improvement of barrier properties through the inclusion of whey protein in film composites has previously been reported for several food film forming agents including gelatine, sodium alginate, LM pectin and carboxymethylcellulose (Murillo-Martínez et al., 2011, Wang et al., 2010). The ability of whey proteins to reduce intermolecular spacing due to hydrogen bonding with the film forming agent, subsequent hindrance of water mobility may explain the lowered water vapour permeability in the WPC based films. The lowest WVP was observed in the low residual moisture content thin HSA/WPC and κ-CAR/LBG/WPC films indicating that a combination of water affinity and reduced water mobility due to WPC inclusion may drive WVP.

### 3.5 Colour and optical characteristics

Colour and light transmission properties are of major importance for edible film fabrication as they directly impact appearance and liking of the packaged/coated food product. HSA and κ-CAR/LBG based edible films had higher L* compared to the other resulting films which could be attributed to their lower solids contents and subsequently lower thicknesses. (Table 1). The addition of WPC induced a significant increase (p < 0.05) of red and yellow hues (Table 4), which confirms previous findings (Ramos et al., 2012) and may be due to the occurrence of maillard chemistry during drying; however, it did not impact the luminosity of the probiotic films.

Film opacity was not significantly (p > 0.05, data not shown) affected by the presence of probiotic cells in line with our previous findings (Soukoulis et al., 2014b), furthermore κ-CAR/LBG and HSA based films exhibited the highest opacity which is presumably due to the lower solids contents of the κ-CAR/LBG and HSA based forming solutions. Film opacity significantly (p < 0.05) increased in the presence of WPC.

### 3.6 Tensile and thermo-mechanical characteristics

In general, edible films must possess good mechanical properties (strength to fracture, extensibility) in order to withstand the stress involved under common processing, handling and storage conditions. The major mechanical aspects of probiotic edible films are given in Table 5. Of the polysacchide films HSA, κ-CAR/LBG and PEC exhibited similar mechanical profile i.e. intermediate tensile strength, good elongation properties, and low stiffness, LSA based systems were characterised by high tensile strength, this is presumably due to a lower Mw of the LSA compared to the HSA. Films containing GEL had a high tensile strength, were more extensible and had a higher tensile strength compared to LSA which is presumably due to is protein based network compared to LSA and the other films. From this standpoint, LSA probiotic films may be a less feasible packaging solution in the case where resistance to high mechanical stresses due to product processing and handling operations is required.

Considering the impact of whey protein, the WPC based film composites had significantly lower mean tensile strength (18.6 vs 96.8 MPa) and lower mean elasticity (6.8 vs 14.8 MPa) than the hydrogel based films.

For the determination of the thermophysical properties of the plasticised, preconditioned films both DSC and DMA analysis was carried out (Table 6). In both analyses, a major peak for stiffness factor (tanδ) and loss module (E″) at low subzero temperatures was observed (−70 to −35 °C), and in several cases a second pronounced (frequency independent) peak at the temperature range of 70–100 °C was detected, representing structural changes taking place due to water evaporation (Soukoulis et al., 2015). DSC thermograms revealed solely the existence of a single second order phase transition at very low temperatures (−80 to −40 °C) corroborating the DMA curves but no phase transition phenomenon was observed in the entire above-zero temperature region (0–150 °C). Similar results have been also reported in previous studies (Denavi et al., 2009, Ogale et al., 2000; Christos Soukoulis et al., 2016). According to Denavi et al. (2009) this is indicative of β-relaxation associated with the presence of plasticiser (i.e. glycerol) rich micro-domains. Regarding the impact of the film components. Biopolymer type had a significant impact on the glass transition values of the films, with the films made with alginates having the highest average T_g_. No significant differences in T_g_ of the PEC, GEL and κ-CAR/LBG films was found therefore the films can be directly compared with the assumption of no major differences in physical state. WPC significantly (p < 0.05) depressed the glass transition temperature (T_g_) which could be attributed to the increased molecular mobility due to the plasticising agents (water and glycerol).

### 3.7 Inactivation of *L. rhamnosus* GG during edible films storage

The inactivation of probiotic cells during storage is governed by several factors including species/strain dependency, storage exposure conditions (temperature, a_w_, RH), presence of protective agents, occurrence of physical state transitions and oxidative damage (Tripathi & Giri, 2014).

Inactivation of *L. rhamnosus GG* during storage was tested at two temperatures (4 and 25 °C) under controlled relative humidity (59% and 54% respectively) as shown in Fig. 2. The inactivation of *L. rhamnsosus GG* followed first order kinetics (Table 2 and Fig. 2) which was in accordance with previous studies (Kanmani and Lim, 2013, Romano et al., 2014, Soukoulis et al., 2016). Both storage conditions and film composition (biopolymer type and WPC supplementation) had a significant impact (p < 0.05) on inactivation rates of *L. rhamnosus GG.* As expected, the inactivation rate of *L. rhamnosus GG* was lower in films stored at chilling conditions (0.099 log CFU day^−1^) than those kept at ambient temperature (0.363 log CFU day^−1^). In previous studies, it has been shown that the dependency of survival rate on storage temperature follows Arrhenius kinetics for systems that do not experience phase transitions throughout storage e.g. glassy to rubbery state (Soukoulis et al., 2014a, Ying et al., 2012). According to the DSC and DMA analysis results, all systems exerted a fairly rubbery physical state (T_g_ << T_storage_) and therefore, storage under controlled RH conditions is presumed not to induce physical state transitions. It is therefore assumed that the enhanced storage stability of *L. rhamnosus* GG under chilling conditions is associated with the slowing of its metabolic activity (Fu & Chen, 2011). In addition, it should be mentioned that low temperatures slow sub-lethal enzymatic and chemical reactions such as lipid oxidation and protein denaturation.

Films fabricated with κ-CAR/LBG or HSA were most effective at maintaining maximal biological activity of the probiotic cells (0.167 and 0.218 log CFU day^−1^ in average) compared to films made of PEC, GEL and LSA (0.251, 0.252 and 0.268 log CFU day^−1^ respective means) this may be explained by the low Tg, and low VWP of the binary system. Although individually these are not significantly different from some other systems together they may partially explain the enhanced stability.

Supplementation of the film forming solutions with WPC resulted in an enhanced *L. rhamnosus GG* storage stability (0.279 and 0.183 log average CFU day^−1^ for systems with and without the addition of WPC respectively). It is well established that proteins can maintain the biological activity of *Lactobacilli* via free radical scavenging which inhibits the peroxidation of membrane lipids, and surface adhesion properties that assist bacterial cells un overcoming physical stresses during storage. In addition, depending on solute composition of the embedding substrate, proteins can modulate their molecular mobility and therefore, the occurrence rate of deteriorative enzymatic and chemical reactions taking place during storage. The bioprotective role of WPC could be primarily associated with its ability to reduce the osmolytic cell injuries arising throughout the dehydration process and their excellent cell adhesion properties as recently confirmed by Burgain et al., 2013a, Burgain et al., 2014. In addition, whey protein hydrolysis compounds (e.g. peptides and aminoacids) naturally occurring in WPC, but also produced by the proteolytic action of *L. rhamnosus* GG, possess very good reducing and free radical scavenging activity preventing lipid autoxidation (Peng, Kong, Xia, & Liu, 2010) and residual lactose may further enhance stability by enhancement of membrane stability by partially mitigating osmotic stress. Focusing on the individual interactions of WPC with the biopolymer substrate, it should be noted that the sodium alginate systems (LSA and HSA) exhibited the highest responsiveness to WPC addition (ca. 2.1-fold improvement of *L. rhamnosus GG* survival) compared to the other film forming agents (survival enhancement was ca. 1.4–1.7-fold for PEC, GEL and κ-CAR/LBG respectively). With the exception of the PEC/WPC system, the *L. rhamnosus* GG survival enhancement throughout storage is in line with the TVC losses during dehydration i.e. alginate systems exerted the highest responsiveness in the presence of WPC (ca. 6–10-fold for LSA and HSA respectively) compared to GEL and κ-CAR/LBG (4- and 2-fold respectively). Sodium alginate has been reported as possessing fair bioadhesive functionality which is driven by the formation of hydrogen bonds (Khutoryanskiy, 2011). In the presence of WPC, anionic polysaccharides can undergo ionotropic gelation, induced by the presence of Ca^2+^ leading to the formation of strong molecular networks that could immobilise and stabalise the bacterial cells (Corona-Hernandez et al., 2013) and may explain enhanced stability in the HSA over the LSA based systems.

**Fig. 5:**
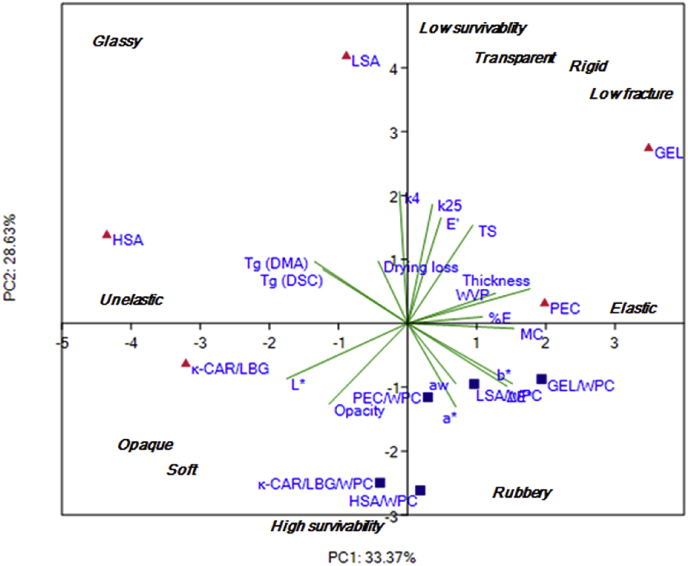
Principal component analysis biplot for the display of the interrelationships between the physicochemical, mechanical and microbiological (total viable counts loss per drying process and storage) properties.

To sum up, the development of edible films as carriers for the delivery of probiotics appears to be a plausible strategy. Although, maintenance of the biological activity of the probiotic cells is the governing parameter for the selection of the substrate compositional aspects other technological parameters such as the mechanical and barrier properties are essential to ensure adequate processibility and shelf life. In an attempt to identify the most promising systems, the obtained experimental dataset (microbiological, mechanical and physicochemical) was subjected to principal components analysis (Fig. 5). The PCA biplot confirmed the complexity of the mechanisms describing the inactivation of *L. rhamnosus* GG throughout storage, in general PCA analysis revealed that κ-CAR/LBG and HSA were the best performing systems and that WPC addition enhanced the biological activity of *L. rhamnosus* GG, these systems are also technologically viable formulations as they have soft, less fracturable and less rigid films. While Tg (glassy to rubbery), moisture content and extensibility were not correlated with survivability; low E′ and low TS and high opacity showed directional correlation with increasing survivability.

## 4 Conclusion

Overall, this work suggests that the inclusion of whey protein isolate increased *L. rhamnosus* GG stability and that cell counts were greatest after drying in pectin + WPC films, and during storage composite carrageenan/locust bean gum/WPC films offered the greatest stability, overall stability in an edible films is therefore proposed to be a composite function of thermal and oxidative stability, in combination with molecular mobility and WVP.

## Figures and Tables

**Table 1:** Compositional aspects of the probiotic film forming solutions.

Edible film	Hydrocolloid (g/100 g)	Whey protein concentrate (g/100 g)	Glycerol (g/100 g)
PEC	4	–	2
LSA	4	–	2
HSA	1	–	0.5
GEL	4	–	2
κ-CAR/LBG	1 (0.8/0.2)	–	0.5
PEC/WPC	2	2	2
LSA/WPC	2	2	2
HSA/WPC	1	2	1.5
GEL/WPC	2	2	2
κ-CAR/LBG/WPC	1 (0.8/0.2)	2	1.5

**Table 2:** Inactivation rates of *L. rhamnosus* GG during storage at chilling (4 °C) and room (25 °C) temperature conditions at controlled relative humidity and estimated shelf life (day) (R^2^ indicates squared correlation coefficient).

Edible film	k_4 °C_ (R^2^)	Shelf-life 4 °C	k_25 °C_ (R^2^)	Shelf-life 25 °C
PEC	0.124 ± 0.010^c^ (0.86)	9	0.424 ± 0.034^b^ (0.99)	3
LSA	0.223 ± 0.018^d^ (0.96)	10	0.470 ± 0.038^c^ (0.98)	5
HSA	0.120 ± 0.010^c^ (0.89)	27	0.397 ± 0.032^b^ (0.95)	8
GEL	0.130 ± 0.010^c^ (0.97)	26	0.493 ± 0.039^c^ (0.99)	7
κ-CAR/LBG	0.085 ± 0.007^b^ (0.95)	39	0.330 ± 0.026^a^ (0.99)	10
PEC/WPC	0.073 ± 0.006^b^ (0.88)	60	0.386 ± 0.031^b^ (0.98)	11
LSA/WPC	0.080 ± 0.003^b^ (0.96)	39	0.301 ± 0.024^a^ (0.99)	10
HSA/WPC	0.041 ± 0.003^a^ (0.96)	99	0.314 ± 0.025^a^ (0.92)	13
GEL/WPC	0.074 ± 0.005^b^ (0.98)	50	0.311 ± 0.018^a^ (0.99)	12
κ-CAR/LBG/WPC	0.047 ± 0.001^a^ (0.85)	70	0.205 ± 0.015^a^ (0.99)	16

Numbers with different superscript letters are significantly different (p < 0.05).

**Table 3:** Residual water content, water activity and thickness of edible films containing *L. rhamnosus* GG. Water content and thickness was measured prior to preconditioning, water activity was measured after preconditioning at 54% RH.

Edible film	Residual water content (g/100 g)	Water activity a_W_	Thickness (μm)
PEC	8.04 ± 0.62^d^	0.53 ± 0.01^a^	120 ± 20^b^
LSA	5.91 ± 0.57^bc^	0.53 ± 0.00^a^	130 ± 20^b^
HSA	2.75 ± 0.33^a^	0.53 ± 0.01^a^	40 ± 10^a^
GEL	5.98 ± 0.13^b^	0.53 ± 0.00^a^	140 ± 20^b^
κ-CAR/LBG	2.44 ± 0.18^a^	0.53 ± 0.00^a^	40 ± 10^a^
PEC/WPC	8.01 ± 0.60^d^	0.53 ± 0.00^a^	110 ± 20^b^
LSA/WPC	7.58 ± 0.03^cd^	0.53 ± 0.01^a^	120 ± 10^b^
HSA/WPC	5.00 ± 0.57^b^	0.52 ± 0.00^a^	90 ± 10^b^
GEL/WPC	6.31 ± 0.67^bcd^	0.53 ± 0.00^a^	120 ± 10^b^
κ-CAR/LBG/WPC	5.13 ± 0.30^b^	0.52 ± 0.00^a^	100 ± 20^b^

Numbers with different superscript letters are significantly different (p < 0.05).

**Table 4:** Colour characteristics and transparency of the probiotic edible films containing *L. rhamnosus* GG.

Edible film	L*	a*	b*	Opacity (mm^−1^)
PEC	87.8 ± 0.22^ab^	−1.11 ± 0.18^def^	12.03 ± 0.43^bcd^	2.15 ± 0.14^b^
LSA	89.4 ± 0.84^bc^	−1.46 ± 0.04^ab^	7.39 ± 0.57^a^	3.31 ± 0.50^bc^
HSA	91.5 ± 0.56^d^	−1.50 ± 0.04^a^	7.22 ± 0.47^a^	5.08 ± 0.31^c^
GEL	87.3 ± 0.82^a^	−1.45 ± 0.11^ab^	11.54 ± 0.66^bc^	0.49 ± 0.05^a^
κ-CAR/LBG	91.2 ± 0.32^d^	−1.28 ± 0.05^bcd^	7.12 ± 0.33^a^	17.21 ± 1.25^f^
PEC/WPC	90.5 ± 0.92^cd^	−1.31 ± 0.04^bcd^	10.04 ± 1.71^b^	9.39 ± 0.54^e^
LSA/WPC	89.1 ± 0.54^bc^	−0.96 ± 0.06^f^	14.11 ± 0.66^d^	6.85 ± 0.06^d^
HSA/WPC	90.5 ± 0.66^cd^	−1.08 ± 0.15^ef^	13.32 ± 1.95^cd^	10.52 ± 0.14^e^
GEL/WPC	88.9 ± 0.42^bc^	−1.23 ± 0.11^cde^	12.13 ± 0.86^bcd^	2.72 ± 0.31^b^
κ-CAR/LBG/WPC	90.4 ± 1.22^cd^	−1.35 ± 0.08^abc^	9.86 ± 0.27^b^	9.96 ± 0.27^e^

Numbers with different superscript letters are significantly different (p < 0.05).

**Table 5:** Mechanical properties of edible films containing *L. rhamnosus* GG.

Edible film	Tensile strength (MPa)	Elongation (%)	Young's modulus (E) (MPa)
PEC	23.1 ± 1.7^de^	52.5 ± 4.7^f^	0.8 ± 0.0^ab^
LSA	133.8 ± 16.2^g^	8.2 ± 0.9^a^	44.9 ± 1.5^h^
HSA	16.5 ± 2.3^c^	33.3 ± 2.8^d^	1.3 ± 0.4^c^
GEL	291.1 ± 38.4^h^	90.2 ± 3.2^g^	24.4 ± 2.0^g^
κ-CAR/LBG	19.6 ± 1.1^cd^	44.1 ± 3.7^ef^	2.5 ± 0.1^e^
PEC/WPC	10.8 ± 0.6^b^	22.9 ± 3.0^b^	1.9 ± 0.1^d^
LSA/WPC	26.8 ± 0.3^e^	23.7 ± 1.5^bc^	17.2 ± 0.3^f^
HSA/WPC	8.7 ± 0.7^a^	28.3 ± 3.2^cd^	0.7 ± 0.0^a^
GEL/WPC	38.2 ± 2.5^f^	82.7 ± 6.2^g^	13.3 ± 0.9^f^
κ-CAR/LBG/WPC	8.5 ± 0.8^a^	40.5 ± 2.9^e^	0.9 ± 0.0^b^

Numbers with different superscript letters are significantly different (p < 0.05).

**Table 6:** Thermophysical properties of the probiotic edible films containing *L. rhamnosus* GG.

Edible film	DSC		DMA
	Glass transition temperature T_g_ (°C)	Change in specific heat capacity ΔC_P_ (kJ/mol*K)	Glass transition temperature T_g_ (°C)
PEC	−66.1 ± 1.4^cd^	0.533 ± 0.034^b^	−57.3 ± 0.8^c^
LSA	−63.0 ± 1.9^d^	0.489 ± 0.037^ab^	−49.6 ± 4.9^b^
HSA	−45.2 ± 0.1^e^	0.529 ± 0.007^b^	−36.4 ± 0.7^a^
GEL	−69.0 ± 0.8^cb^	0.405 ± 0.013^a^	−62.9 ± 1.1^d^
κ-CAR/LBG	−66.6 ± 0.5^cd^	0.376 ± 0.000^a^	−53.1 ± 0.9^bc^
PEC/WPC	−72.1 ± 1.8^ab^	0.463 ± 0.034^ab^	−68.1 ± 4.0^e^
LSA/WPC	−63.5 ± 1.6^d^	0.483 ± 0.012^ab^	−56.5 ± 2.8^c^
HSA/WPC	−65.0 ± 0.8^cd^	0.370 ± 0.031^a^	−55.0 ± 2.3^c^
GEL/WPC	−72.0 ± 0.7^ab^	0.402 ± 0.007^a^	−68.7 ± 1.8^e^
κ-CAR/LBG/WPC	−75.4 ± 0.2^a^	0.392 ± 0.022^a^	−69.0 ± 2.8^e^

Numbers with different superscript letters are significantly different (p < 0.05).
