# Supplementary material for: Stability of Lactobacillus rhamnosus GG incorporated in edible films: Impact of anionic biopolymers and whey protein concentrate
Source: Food Hydrocoll. 2017 Sep;70:345–55. doi: 10.1016/j.foodhyd.2017.04.014 (PMC5429391; doi:10.1016/j.foodhyd.2017.04.014)
Supplement: Supplementary file 1 [file mmc1.docx]

APPENDIX’S FIGURE

FIGURE A.1 Indicative DMA spectra of probiotic films with (green/light) or without (blue/dark) whey protein concentrate. a: κ-CAR/LBG, b: LSA.
